# Systemic Immune Modulation by Gastrointestinal Nematodes

**DOI:** 10.1146/annurev-immunol-090222-101331

**Published:** 2024-06-14

**Authors:** Darshan N. Kasal, Lindsey M. Warner, Astra S. Bryant, Elia Tait Wojno, Jakob von Moltke

**Affiliations:** 1 Department of Immunology, University of Washington, Seattle, Washington, USA;; 2 Department of Physiology and Biophysics, University of Washington, Seattle, Washington, USA

**Keywords:** type 2 immunity, mucosal immunity, helminth infection, host–parasite interaction

## Abstract

Gastrointestinal nematode (GIN) infection has applied significant evolutionary pressure to the mammalian immune system and remains a global economic and human health burden. Upon infection, type 2 immune sentinels activate a common antihelminth response that mobilizes and remodels the intestinal tissue for effector function; however, there is growing appreciation of the impact GIN infection also has on the distal tissue immune state. Indeed, this effect is observed even in tissues through which GINs never transit. This review highlights how GIN infection modulates systemic immunity through (*a*) induction of host resistance and tolerance responses, (*b*) secretion of immunomodulatory products, and (*c*) interaction with the intestinal microbiome. It also discusses the direct consequences that changes to distal tissue immunity can have for concurrent and subsequent infection, chronic noncommunicable diseases, and vaccination efficacy.

## INTRODUCTION

Numerous nematodes have evolved to parasitize the gastrointestinal tract of humans and other mammals. These gastrointestinal nematodes (GINs) include soil-transmitted helminths (hookworms, whipworms, ascarids, and the rodent parasite *Heligmosomoides polygyrus*), *Strongyloides* spp., *Trichinella* spp., and others. Although usually nonlethal, chronic and recurring GIN infections account for enormous economic and health burdens. Soil-transmitted helminths alone persistently and recurrently infect an estimated 1.5 billion people (1). However, the parasitic burden is disproportionately distributed, with higher prevalence in sanitation-poor tropical/subtropical regions and diminished prevalence in postindustrialized societies (2).

**Figure 1:**
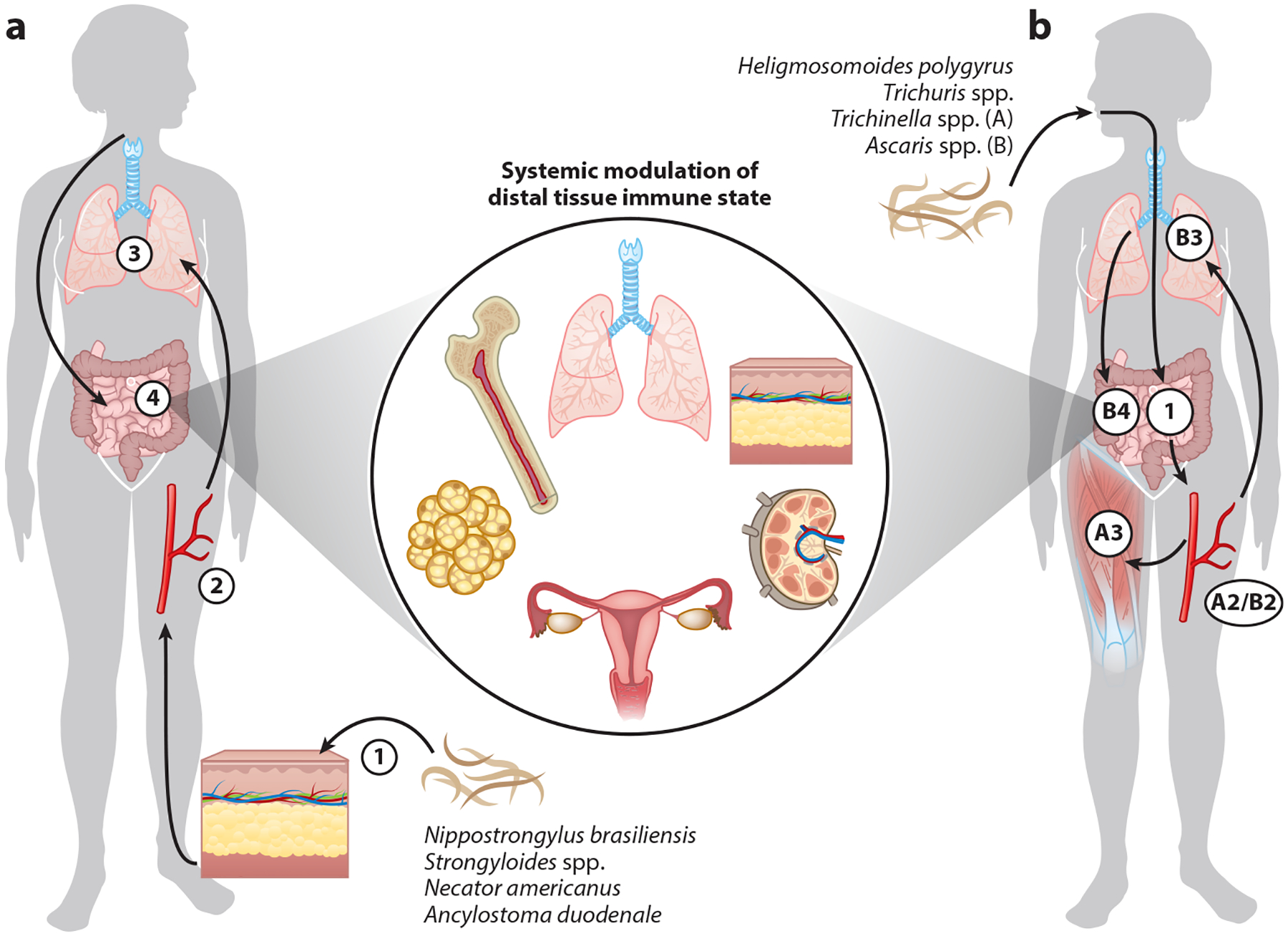
Systemic modulation of distal tissue immune state by gastrointestinal nematodes despite diverse infectious life cycles. (*a*) Larvae of skin-penetrating gastrointestinal nematodes (GINs) transit through the skin (①), circulation (②), and lung (③) before arriving in the duodenum of the small intestine (④). (*b*) Orally ingested GINs arrive in (①) the small intestine before diverging in infectious life cycle. *Heligmosomoides polygyrus* larvae mature and remain in the proximal small intestine, *Trichuris* spp. larvae colonize the colon, and *Trichinella* and *Ascaris* spp. larvae exit the gut into circulation (A2/B2). Then, *Trichinella* spp. encyst in muscles (A3), while *Ascaris* spp. transit through the lung (B3) before returning to the small intestine (B4). The immune state of many tissues is altered by these infections, including tissues through which helminths did not transit.

**Figure 2:**
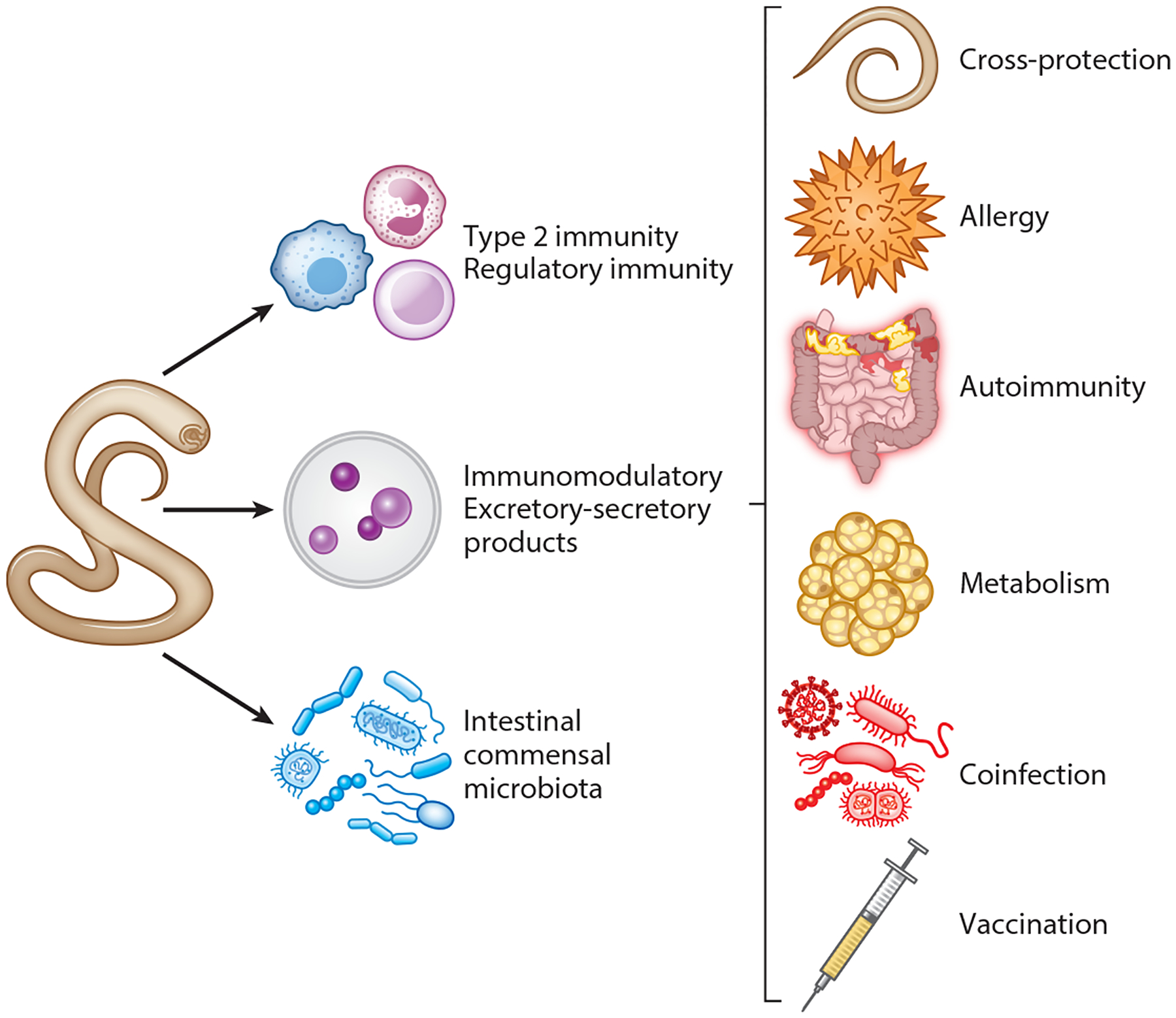
Gastrointestinal nematode (GIN) infection affects host systemic immunity. Host response to infection, chronic noncommunicable disease, and vaccination are altered by immune resistance and tolerance responses to GINs, secretion of immunomodulatory products by GINs, and GIN interaction with the intestinal microbiota.

Although all GINs colonize the intestinal tract, their life cycles both within and outside mammalian hosts are enormously diverse (Figure 1). Hookworm and *Strongyloides* spp. larvae penetrate the skin and pass through the lung before reaching the small intestine. Whipworm eggs, ascarid eggs, and *Trichinella* larvae are directly ingested: Whipworm adults colonize the colon; ascarid larvae leave the small intestine, migrating through the lungs before returning to the small intestine; and *Trichinella* adults colonize the small intestine, while their larvae migrate to and encyst in muscle. Notably, in all cases the immune effects of GIN infection are not restricted to the tissues they transit through or colonize (Figure 1). Host immunity, helminth-derived immunomodulatory excretory-secretory (ES) products, and infection-induced alterations to the microbiome all contribute to the remodeling of distal immune environments, such that GIN infection has a systemic impact on concurrent or subsequent immune responses. Where GINs are endemic, infection with multiple helminth species and microbial pathogens is possible (3), and epidemiological evidence suggests an inverse correlation between GIN infection and the occurrence of allergy, autoimmunity, and metabolic dysfunction (2). Furthermore, helminth infection can influence the vaccination response. Thus, in this review we highlight recent studies describing how host–parasite interactions during enteric nematode infection modulate the immunological state of numerous peripheral tissues and how that, in turn, can influence host immunity to various immunological diseases (Figure 2).

## OVERVIEW OF IMMUNITY TO GASTROINTESTINAL NEMATODES

**Figure 3:**
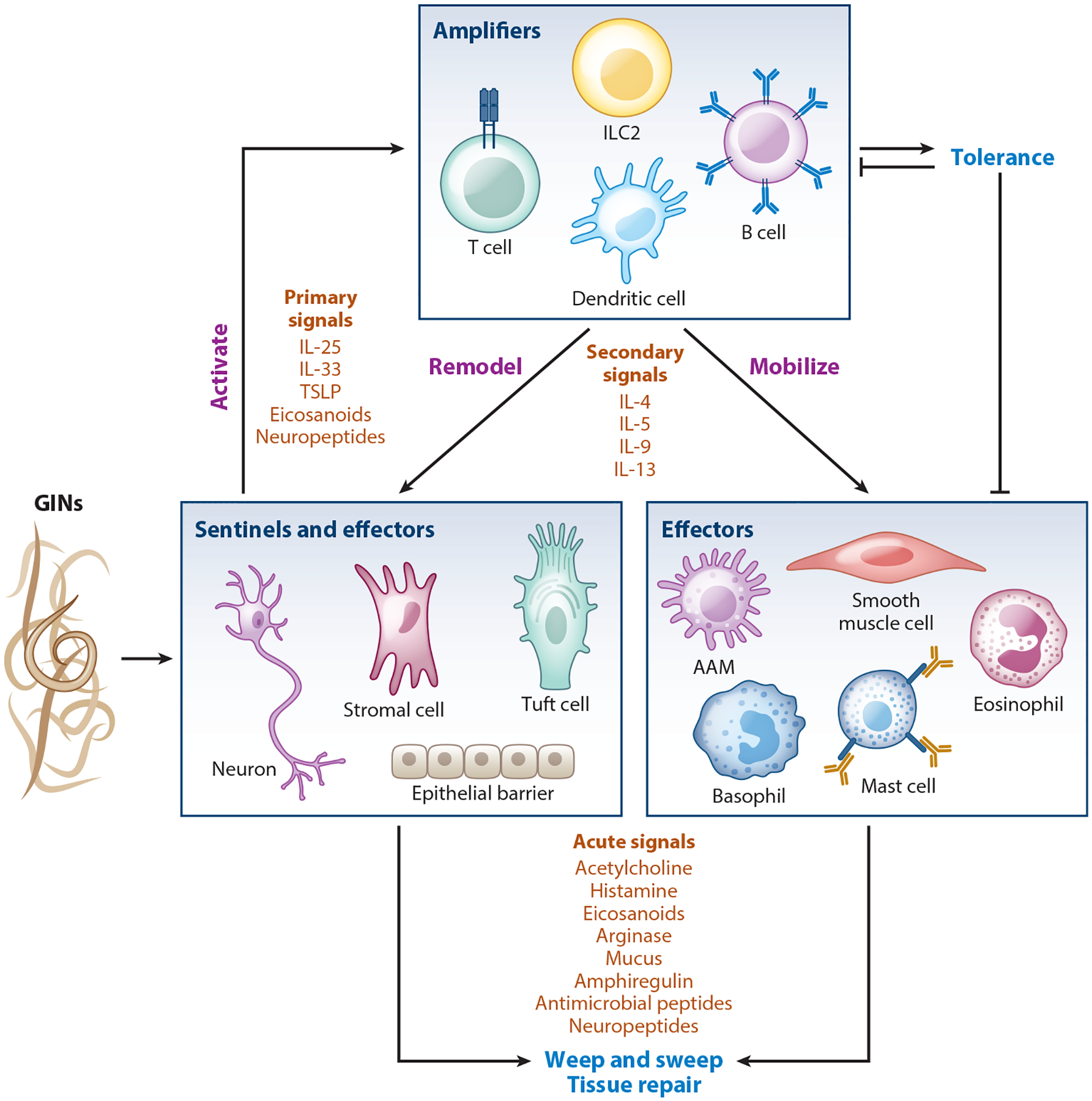
Overview of type 2 immune response to gastrointestinal nematode (GIN) infection. Tissue-specific sentinels of type 2 immunity sense GINs and release primary signals that activate the immune amplifiers. These cells then release secondary signals that mobilize hematopoietic effector cells and remodel sentinel cells as well as other, nonhematopoietic cells into immune effectors. After mobilization and remodeling are complete, these effectors release signals that mediate the “weep and sweep” response to clear GIN infection and repair damaged tissue. During chronic infection, tolerogenic mechanisms induced by the host act as a break on amplifier cells to limit pathological type 2 immune responses. GINs have also evolved to interfere with the host immune response by secreting products that can induce tolerogenic responses or nullify sentinel cell function and activating signals. Abbreviations: AAM, alternatively activated macrophage; ILC2, group 2 innate lymphoid cell; TSLP, thymic stromal lymphopoietin.

Immunity to helminth infection results from a coordinated innate and adaptive immune response distinct from the humoral and cellular responses activated during viral, bacterial, fungal, or even unicellular parasitic infection. Collectively termed type 2 immunity, this response is also triggered by allergens and certain toxins (4). Type 2 immunity relies predominantly on IL-4, IL-5, IL-9, IL-13, IL-25, IL-33, and thymic stromal lymphopoietin (TSLP), with important contributions from lipid-derived eicosanoids (e.g., prostaglandin D_2_, cysteinyl leukotrienes) and neuropeptides (e.g., neuromedin U, calcitonin gene–related peptide) (5) (Figure 3). On the cellular level, key players include mast cells, eosinophils, basophils, alternatively activated macrophages (AAMs), group 2 innate lymphoid cells (ILC2s), type 2 helper T (Th2) cells, and IgE- and IgG1-producing B cells. Central to antihelminth immunity are ILC2s and Th2 cells, which respond to tissue-specific activating signals and secrete type 2 cytokines responsible for coordinating, remodeling, and mobilizing the other components of the response. In this section, we provide a brief overview of immunity to GIN infection, which has been extensively reviewed elsewhere (5).

### Innate Sensing and Inflammation

Detection is the obligate first step of any immune response, yet how it works during GIN infection remains an area of active investigation. In many infections, immune cells directly detect the pathogen (e.g., macrophages sense bacteria or viruses via pattern recognition receptors), but there is limited evidence that this process occurs during GIN infection. Tissue-resident ILC2s, for example, are among the first immune cells activated upon GIN infection, but they respond only to host-derived cytokines, lipids, and neuropeptides. Nonhematopoietic cells such as the epithelium, neurons, and stromal cells are the dominant source of these ILC2-activating signals and thus serve as sentinels for type 2 immunity (6). Epithelial and adventitial stromal cell–derived IL-33 is central to ILC2 activation for GINs with lung-infective stages (7, 8). Although these cells constitutively express IL-33, it is normally sequestered in the nucleus (9). The exact mechanisms that mediate IL-33 release remain unclear, but cell lysis or at least cell stress is required. Recent studies implicate the formation of noncanonical gasdermin D or C pores (10, 11) as well as activation of a RIPK1/caspase-8 “ripoptosome” (12) and/or perforin-2 pores (13) in IL-33 release. Helminths may activate these mechanisms or cause IL-33 release by mechanically lysing cells during their migration through tissue. In addition to IL-33, activation of lung ILC2s during hookworm infection requires LTC_4_ and its metabolite LTD_4_, which synergize with IL-33 signaling by activating NFAT (14, 15). Which cells make LTC_4_ and how hookworm infection induces lung LTC_4_ synthesis remain unknown.

In the intestine, IL-25 is more important than IL-33 for ILC2 activation (16) and synergizes with leukotrienes and the neuropeptide NMU, both of which can activate NFAT (17–20). Epithelial tuft cells are the exclusive source of IL-25 in both the small intestine and colon, and they are also the physiologically relevant source of LTC_4_ during GIN infection (20–22). Moreover, tuft cells serve as sentinels that detect GINs in the lumen. Tuft and type II taste receptor cells in the tongue share a master regulator (POU2F3) and transcriptional signature; indeed, tuft cells have chemosensing functions that are mediated by the same intracellular pathway as bitter, sweet, and umami taste (23). Specifically, tuft cells express the calcium-gated cation channel TRPM5, which is required for detection of both helminths and intestine-dwelling *Tritrichomonas* protists (21, 24). How GINs induce the calcium flux required to open TRPM5 remains uncertain. Canonical bitter taste receptors expressed by tuft cells potentiate type 2 immunity during *Trichinella spiralis* infection (25) but do not mediate initial sensing. Tuft cells also express SUCNR1, the receptor for extracellular succinate (26), through which they detect *Tritrichomonas* protists and bacterial dysbiosis, but SUCNR1 is completely dispensable during GIN infection (26, 27). Perhaps tuft cells use a different receptor to sense GIN-derived ligands or detect GIN migration through and along the epithelium. It remains unclear whether tuft cells can still sense GINs if they penetrate the intestinal tissue, and they have not yet been implicated in sensing large intestine–resident helminths, such as *Trichuris* spp. Tuft cells are also present in the tracheal and nasal epithelium, but they do not contribute to the sensing of GINs (20), which transit predominantly through the distal lung.

In addition to activating tuft cells, many GINs cause physical damage in the intestine, and the release of cellular contents may be an important signal for innate type 2 immunity. IL-33 expression is modest in the intestinal epithelium of uninfected mice, but ATP released during GIN infection is converted to adenosine and the A2B adenosine receptor on epithelial cells promotes ILC2 activation, perhaps via induction of IL-33 (28, 29).

Once activated, ILC2s are a critical early source of IL-5, IL-13, and IL-9, which collectively amplify the type 2 response by remodeling tissue and mobilizing effector cells (30). Even prior to infection, ILC2s in all tissues constitutively secrete IL-5, which sustains homeostatic eosinophil production in the bone marrow (31, 32). During infection, IL-13 induces eotaxin production, thereby recruiting eosinophils into the tissue, where they are maintained by locally produced IL-5 and secrete factors that contribute to type 2 inflammation (33). IL-13 also drives AAM differentiation and monocyte recruitment (34). IL-9, meanwhile, supports mast cell growth and function. Mast cells are best known for their functions following IgE cross-linking (discussed in the section titled Effector Functions, below), but they also contribute to innate inflammation, including the response to IL-33, neuropeptides, and perhaps helminth-secreted peptides or proteases (35, 36). Finally, basophils promote protection during primary infection with *T. spiralis* and *Trichuris muris* and during secondary infection with *Nippostrongylus brasiliensis* and *H. polygyrus* (37, 38). During infection, basophils are activated by IL-3, IL-33, TSLP, FcεRIα cross-linking, and Notch signaling to produce IL-4, bioactive lipids, amines, and proteases (37–39). However, the precise mechanisms by which mast cells and basophils contribute to type 2 immunity during infection with different helminths remain incompletely defined.

### Establishing Adaptive Type 2 Immunity

Th2 cells and IgE- or IgG1-secreting B cells are required for antihelminth immunity, but how these cells are primed and activated remains poorly understood. Th2 cell priming requires IRF4-expressing dendritic cells (DCs) and IL-4 (40–42), yet DCs do not express IL-4. Eosinophils, basophils, mast cells, natural killer T cells, and γδ T cells are all capable of IL-4 production; however, none are individually required for Th2 cell priming (43). Instead, T cell–intrinsic IL-4 is sufficient for Th2 cell priming (44, 45), suggesting that DCs use signals other than IL-4 to direct Th2 differentiation. Many factors have been implicated, including antigen dose, DC expression of OX40L, and lack of type 1 cytokines, but a unifying model remains out of reach (43). It is also unclear what signals first instruct DCs to migrate to lymph nodes and direct Th2 cell differentiation, although several cytokines, such as IL-13, IL-33, and TSLP, have been implicated (43). Following skin allergen challenge, DC migration to lymph nodes requires CCR8 and neuron-derived substance P, but these factors have not been tested in GIN infection (46, 47). Whether ILC2s are involved in Th2 cell priming also remains unresolved and is likely context dependent (34, 48–51). Lastly, primed Th2 cells require a “licensing” event in the tissue to activate their effector functions. Specialized antigen-presenting myeloid subsets (52), the tissue-derived signals that activate ILC2s (53), and the epidermal growth factor receptor (54) all contribute to this licensing.

On the B cell side, IgE and IgG1 class switching similarly requires IL-4 and perhaps IL-13; however, the underlying mechanisms remain unclear, although T follicular helper cells are required (55). Notably, there are numerous examples where Th2 cell differentiation or function is nearly absent but IgE production remains unaltered (42, 53), suggesting that distinct mechanisms can direct Th2 cells and the T follicular helper cells that determine B cell fate.

### Effector Functions

The large size of GINs (in some cases >1 m) presents a unique challenge for immune defense. Bacteria and viruses are small enough to be engulfed and degraded by individual immune cells (e.g., neutrophils), but the entire infected tissue must mount a coordinated effort to expel GINs or prevent their migration. In large enough numbers, swarms of eosinophils can slow or perhaps even kill GINs in the tissue, though little evidence indicates that eosinophils are necessary, let alone sufficient, for protective immunity (56, 57). Arginase expressed by ILC2s and AAMs also contributes both directly and indirectly to helminth restriction (58, 59), while the role of antibodies in antihelminth immunity varies between GIN models and between primary and secondary infection (60). IgE serves primarily to degranulate mast cells and basophils, but the protective effector functions directed by IgG1 are less clear and likely include a combination of direct GIN binding and activation of immune cells via Fc receptors.

The centerpiece of type 2 effector functions, however, is the remodeling of tissue structure and function, with the goal of expelling or confining the helminth. These processes are directed predominantly by IL-4 and IL-13, which signal through a shared receptor consisting of IL-4Rα and IL-13RA1 expressed on all the tissue cells that initially served as type 2 sentinels (epithelial cells, neurons, fibroblasts, etc.), as well as on smooth muscle and many hematopoietic cells. In all these cells, IL-4/IL-13 signaling alters differentiation and function in ways that make the intestine more inhospitable for GINs and prepares the tissue for GIN expulsion. For example, IL-4/IL-13 signaling on intestinal epithelium leads to goblet cell hyperplasia, increased mucus production, changes in antimicrobial peptide expression, and a general increase in turnover (61–64). In the small intestine, but not the colon, IL-4/IL-13 signaling also drives a dramatic tuft cell hyperplasia, suggesting that tuft cells contribute to both the sentinel and effector phases of type 2 immunity in the small intestine (21). ILC2s are the dominant early source of IL-13, followed by Th2 cells that make both IL-4 and IL-13. Basophils, mast cells, eosinophils, and innate-like T cells also add to the total cytokine pool (65).

Once tissue remodeling is complete and effector cells have been mobilized (e.g., mast cells are loaded with IgE), more acute signals drive a worm clearance mechanism sometimes referred to as “weep and sweep.” Acetylcholine is central to this response, as it induces fluid and mucus secretion as well as muscle contraction (66–69). Tuft cells are an important source of acetylcholine, and mice lacking M3 muscarinic acetylcholine receptors have delayed worm clearance (70). Histamine and prostaglandin E_2_ released from mast cells also activate acute fluid secretion, although the requirement for mast cells in protective immunity varies between GIN models (71, 72).

Given the damage that worms cause, tissue repair is another critical type 2 effector function. IL-4 and IL-13 again serve as master regulators by generating AAMs and promoting myofibroblast differentiation (34, 73). AAMs secrete numerous growth factors, while myofibroblasts produce and organize extracellular matrix. Eosinophils have also been implicated in tissue remodeling and repair (74), and ILC2s directly contribute to tissue repair by secreting the epidermal growth factor amphiregulin (75).

In humans, chronic GIN infection is often associated with IL-10, regulatory T cells (Tregs), and other markers of tolerogenic responses (76). As most mouse models of GIN infection are acutely cleared, tolerogenic processes are perhaps underappreciated; however, *H. polygyrus*, a model for chronic GIN infection, robustly induces Tregs. Here, Treg induction is mutually beneficial, constraining type 2 immunity to limit pathology during long-term infection while also supporting helminth survival. Collectively, type 2 immune responses can provide sterilizing immunity in mouse models of primary and secondary GIN infection, but in humans, GIN infection is often chronic and protection against reinfection following drug therapy is often transient. Unfortunately, when type 2 immune responses are misdirected at allergens, they cause debilitating and even life-threatening symptoms, and when the wound healing process is hyperactivated or continues for too long, it leads to fibrosis that impairs organ function.

## INTESTINAL HELMINTH-INDUCED TYPE 2 IMMUNITY IN PERIPHERAL TISSUES

Local intestinal immune responses promote worm clearance and host adaptation; however, helminth-induced immunity is not intestinally restricted, even when the worm does not leave the intestine (Figure 1). Numerous studies have documented a systemic type 2 immune response resulting in long-term changes to the immune compartment of distal uninfected tissues, such as the skin, lung, and adipose tissue. Enteric infection with *H. polygyrus* triggers a type 2 immune response in the spleen and lymphoproliferation, resulting in splenomegaly (77–79). Moreover, *H. polygyrus* infection leads to the systemic accumulation of IL-4^+^ Th2 cells and eosinophils in nondraining secondary lymphoid organs and uninfected peripheral tissues (e.g., peritoneal cavity, liver, lung) just 2 weeks postinfection. Notably, following *H. polygyrus* or hookworm infection, the peritoneal cavity represents a dominant reservoir for Th2 cells (77), which may be retained by infection-regulated cell adhesion marker expression (80) and are sufficient to confer protective immunity upon transfer (81). While type 2 immune responses in secondary lymphoid organs diminish upon resolution of infection (82), Th2 cells in peripheral tissues persist long after helminth clearance and acquire tissue-resident and innate-like properties (82–86). This section reviews recent studies investigating how GIN infection modulates distal tissue immune state; the impact of infection on host immunity to varied diseases is discussed further in the section titled Impact of Gastrointestinal Nematode Infection on Host Immunity, below.

### Bone Marrow and Blood

A hallmark of patent helminth infection is blood eosinophilia (87). Following GIN infection, serum IL-5 increases (88), inducing IL-5Rα^+^ eosinophil precursor expansion in the bone marrow (84, 89, 90). Subsequently, levels of circulating blood eosinophils increase, followed by IL-5-dependent eosinophilia in the lymph, peritoneal and pleural cavities, and peripheral organs (77, 89, 90). GIN infection also elevates serum IL-13, supporting the recruitment of eosinophils to distal tissues (84, 88). Thus, an increase in serum IL-5/IL-13 and subsequent systemic eosinophilia are a direct consequence of rapid ILC2 activation by helminth infection.

Beyond the immediate effects of host-derived cytokines, infection by helminths or exposure solely to their ES products may effect long-term changes in the bone marrow by inducing trained immunity, defined as epigenetic reprogramming in innate immune cells that leads to an altered responsiveness to subsequent unrelated insults (84, 91, 92). Infection with *Strongyloides venezuelensis* leads to a sustained increase in bone marrow eosinophil precursors after resolution of infection (84). Remarkably, transfer of long-term hematopoietic stem cells from previously infected mice into naïve recipients leads to a greater accumulation of eosinophils in the duodenal but not ileal lamina propria, particularly if the tissue is first primed by *S. venezuelensis* infection. Separately, subcutaneous injection of liver fluke *Fasciola hepatica* ES products biases the myeloid development potential of bone marrow precursors toward anti-inflammatory monocytes, and this effect is transferable via transplantation of bone marrow or long-term hematopoietic stem cells (91, 92). Whether all GIN infections or ES products from model GINs with known immunomodulatory capabilities, such as *H. polygyrus* (discussed in the section titled Immunoregulation by Helminths, below), are capable of inducing trained immunity in the bone marrow remains to be determined.

### Lung

Many human pathogenic and mouse model GINs have larval stages that migrate to the intestines via the blood and lung (93) (Figure 1). Robust induction of lung type 2 immunity occurs following infection with *N. brasiliensis*, *S. venezuelensis*, and *Ascaris suum* (3, 83, 88, 94, 95). However, enteric helminth infection also induces a type 2 immune response in the lung, even when infection is limited to the intestine. The intestinal stage of *N. brasiliensis* induces interorgan trafficking of gut-derived ILC2s to the lung (88, 96, 97). Notably, administration of the tuft cell–derived cytokine IL-25 is sufficient to promote lung migration of gut-derived ILC2s (96, 97); conversely, IL-25 and its receptor are required for ILC2 migration during *N. brasiliensis* infection (88, 97). Restriction of the infective stage of *N. brasiliensis* to the intestine via oral gavage of adult worms (88), or infection with the intestine-restricted helminth *T. spiralis* (98), further established that enteric infection alone promotes gut-derived ILC2 migration to the lung. Notably, *T. spiralis* or *H. polygyrus* infection results in goblet cell hyperplasia and a sustained increase in mucin MUC5B and MUC5AC secretion in the lung (98), which requires IL-13 and ILC2s but not adaptive immunity. Whether local IL-13 secretion by gut-derived ILC2s is necessary for lung goblet cell hyperplasia and mucin production or whether elevated serum IL-13 (84, 88) is sufficient remains to be determined. Importantly, the induction of lung type 2 immunity by enteric helminth infection is not exclusive to innate immune cells. Th2 cells and Tregs accumulate in the lung following *H. polygyrus* infection (77, 99, 100), and Th2 cells acquire innate-like properties, responding to secondary infection in an antigen-independent and IL-33-dependent manner (83).

### Skin

Similar to the lung, several GINs pass through the skin as a part of their infectious life cycle (Figure 1). Nevertheless, *H. polygyrus* infection leads to skin eosinophilia and an increase in skin Th2 cells, which acquire a tissue-resident phenotype, are sustained after worm clearance, and respond to *H. polygyrus* antigen restimulation (85). Whereas Th2 cells and Tregs are robustly induced in the mesenteric lymph nodes during *H. polygyrus* infection, they are absent from skin-draining lymph nodes (101). Instead, Th2 cell appearance in the skin is associated with the upregulation of skin-homing receptors CCR4 and CCR10 on mesenteric lymph node T cells, suggesting that Th2 cells are primed for systemic migration in the mesenteric lymph nodes.

Intestinal helminth infection results in a pronounced hyperplasia of gut-draining mesenteric lymph nodes in part through redistribution of naïve lymphocytes in an antigen-independent manner (101, 102). Conversely, during chronic helminth infection, skin-draining lymph nodes (e.g., popliteal, axillary, inguinal) undergo profound hypoplasia, which is reversed upon worm clearance. Thus, sequestration of lymphocytes in the mesenteric lymph nodes during ongoing intestinal helminth infection can limit peripheral lymph node responses (85, 101–103).

### White Adipose Tissue

At homeostasis, type 2 immune cells, including eosinophils, ILC2s, and AAMs, are enriched in white adipose tissue (WAT), where they regulate host metabolic function (32, 104, 105). Strikingly, following infection with *N. brasiliensis* or *H. polygyrus*, eosinophils and tissue-resident Th2 cells increase in WAT depots, such as the perigonadal WAT and mesenteric WAT, where they persist after worm clearance (32, 86, 104). Specific differences in the expansion of type 2 immunity at distinct WAT depots may exist. While *H. polygyrus* infection induces mesenteric WAT remodeling and augmented IL-33 and collagen production, these changes are not observed in the perigonadal WAT (86). Whether these differences are tissue intrinsic or determined by proximity to the site of infection remains unclear.

### Female Reproductive Tract

Intestinal helminth infection in humans is associated with alterations to fecundity (106) and type 2 cytokine expression in the female reproductive tract (FRT) (107). In mice, infection with *N. brasiliensis* results in ILC2 and eosinophil recruitment to the FRT and increased IL-4, IL-5, and IL-33 in the tissue and/or lavage (108). Notably, eosinophil recruitment to the FRT in this context is IL-33 dependent but *Il4ra* independent.

## IMMUNOREGULATION BY HELMINTHS

Given the ability of helminths to colonize their hosts for years or even decades, it is unsurprising that they have evolved numerous strategies to evade and manipulate host immune defense.

All helminths secrete a diverse mixture of proteins, lipids, small molecules, and extracellular vesicles (EVs), referred to as ES products, and numerous studies have demonstrated that such ES products from human and murine GINs are sufficient to activate or modify host immunity. Immunoregulation by helminths has been comprehensively reviewed elsewhere (109). In this section, we highlight several recent discoveries in this field; we review how helminth ES products modulate systemic immunity to disease in the section titled Impact of Gastrointestinal Nematode Infection on Host Immunity, below.

### Cytokine Mimics and Inhibitors

The manipulation of cytokine signaling represents a direct and potent mechanism for regulation of host immunity. HES (ES products from *H. polygyrus*), which is associated with accumulation of Tregs, has TGF-β-like activity (110). TGF-β is an important factor for the differentiation of Tregs, and HES is sufficient to generate FOXP3^+^ Tregs when paired with T cell receptor stimulation in vitro. Screening of HES fractions identified the protein responsible for this activity: Hp-TGM, a structurally distinct TGF-β mimic with TGF-β receptor–binding capability encoded by *H. polygyrus* (111).

HES also blocks IL-33 release, and screening of HES fractions identified two *H. polygyrus* proteins, HpARI and HpBARI, that directly inhibit IL-33 function (112, 113). HpARI binds IL-33 and DNA, allowing it to both limit release of IL-33 from cell nuclei and prevent binding to the IL-33 receptor subunit ST2. HpBARI, in contrast, binds ST2 to prevent interactions with IL-33. Consistent with the rodent tropism of *H. polygyrus*, it appears that HpBARI evolved to specifically bind rodent ST2. Surprisingly, *H. polygyrus* also encodes an HpBARI homolog (HpBARI_Hom2) that, unlike HpBARI, has high affinity for human ST2. HpARI, HpBARI, Hp-TGM, and several Hp-TGM homologs all contain multiple complement control protein-like domains that may provide plasticity for the evolution of host-targeting effectors (113, 114). Whether other GINs encode similar and/or distinct cytokine mimics or inhibitors has not been well characterized, but p43, the dominant protein in *T. muris* ES products, is structurally related to the host-encoded IL-13 decoy receptor IL-13RA2 and blocks the effects of IL-13 in vitro (115).

### Extracellular Vesicles

Release and uptake of EVs is a common mechanism of cell–cell communication (116). An analysis of HES revealed the presence of EVs derived predominantly from the *H. polygyrus* intestine (117). These EVs are taken up by host myeloid and epithelial cells and contain *H. polygyrus*–derived RNAs, including microRNAs predicted to target host RNAs (117, 118). Treating macrophages with EVs broadly inhibits their phenotypic differentiation and specifically downregulates ST2. Culturing colon tissue with EVs from *N. brasiliensis*, but not *T. muris*, upregulates IL-10 and suppresses proinflammatory cytokine expression (119). Lastly, immunizing mice with *H. polygyrus*–derived EVs and alum is sufficient to protect against subsequent *H. polygyrus* infection (118). Thus, EVs contain numerous immunomodulatory factors, and further study will be needed to dissect their effects.

### Epithelial Manipulation

As described above, epithelial remodeling is a critical component of host defense against helminth infection, and GINs have evolved effectors to directly interfere in this process. Two recent studies reported that HES reprograms the intestinal epithelium (120, 121). HES-treated organoids become cystic, lose canonical stem cell markers, upregulate a fetal-like transcriptional program, and cannot induce tuft cell hyperplasia in response to IL-4/IL-13 treatment. *H. polygyrus* infection or HES injection also dampens epithelial remodeling in vivo, leading instead to broad induction of Clusterin expression, which indicates the possible emergence of so-called revival stem cells (121, 122). Epithelial reprogramming also occurs in the crypts above the *H. polygyrus* granuloma, but this effect is transient and does not occur in IFN-γ^−/−^ mice (123). It appears, therefore, that the host induces a localized repair program during the early granuloma stages of *H. polygyrus* infection, while at later stages of infection HES leads to a broader reprogramming of the epithelium to support chronic luminal colonization. Notably, this reprogramming does not occur during infection with *N. brasiliensis*, which does not invade the tissue and is rapidly cleared from the intestine (121). Future studies will likely identify the specific factor(s) in HES that mediate this reprogramming.

### Other Immunomodulatory Proteins

Although HES has been best characterized and most extensively studied, there are several other examples of ES proteins with immunoregulatory function. For example, GINs secrete several acetylcholinesterases that might interfere with acetylcholine-induced epithelial secretion and smooth muscle contraction (124). *N. brasiliensis* secretes a DNase capable of degrading neutrophil extracellular traps that may impede its skin phase of infection (125). The AIP1 and AIP2 proteins secreted by the canine hookworm *Ancylostoma caninum* are also sufficient to suppress inflammation (126, 127). In the case of AIP2, this process requires modulation of Tregs in the intestine-draining mesenteric lymph nodes. Given the size of helminth genomes and the complexity of ES products, it is likely that many additional immunoregulatory factors remain to be discovered.

## GENETIC MANIPULATION OF HELMINTHS

Functional genomics in model bacterial and viral systems has proved invaluable in the effort to understand and manipulate immunological responses directed at these microorganisms. Moreover, mutagenesis has permitted the discovery of strategies employed by microbial pathogens to survive within a host and evade or suppress host detection and elimination mechanisms. Equivalent insights for parasitic worms have remained elusive, largely due to the historical lack of tools for functional genomics in these species. The challenges and recent advances in establishing genetic tools in GINs have been reviewed elsewhere (128–131). Here, we briefly discuss three key techniques for altering gene expression that may enable new insights into GIN-triggered immune responses: transgenesis, mutagenesis, and knockdown.

### Transgenesis in Gastrointestinal Nematodes

The most well-established technique for genetic manipulation of GINs is transgenesis applied to species of the Strongyloididae family, which are unique among GINs in having a free-living adult life stage whose anatomical similarity to the free-living *Caenorhabditis elegans* has facilitated the adaptation of *C. elegans* culturing and genetic modification techniques (128–132). Notably, although *Strongyloides* spp. transmit microinjected transgenes through multiple generations, expression is suppressed after the F1 generation due to active silencing of extrachromosomal DNA. Generation of stable *Strongyloides* spp. transgenic lines requires genomic integration, which is currrently most feasible using the *piggyBac* transposase system in *Strongyloides ratti* (129, 131).

Intragonadal microinjection has been used to generate progeny expressing a range of transgenes (128–131). So far, transgenesis in *Strongyloides* spp. has been used predominantly to investigate the neural basis of host seeking and infection and to understand the genetic mechanisms controlling development and survival of infective larvae (129–131). For functional investigations of how parasites trigger host immunity, a notable recent study assessed the function of CD4^+^ T cells specific for helminth-derived antigens by generating a stable transgenic line of *S. ratti* that expresses an immunodominant CD4^+^ T cell epitope, 2W1S (133).

Beyond the Strongyloididae family, methods of gene transfer that do not rely on intragonadal microinjection have also been used, with varying efficacy. Microparticle bombardment of *Ascaris* embryos can drive transient expression of DNA and RNA constructs (131). Recently, transduction of *N. brasiliensis* infectious third larval stage (iL3) was achieved using a vesicular stomatitis virus G (VSV-G)-pseudotyped lentivirus with successful heritable integration into genomic DNA; nevertheless, expression was silenced in F1 parasitic adults (134). Optimization of transgene constructs (e.g., inclusion of endogenous regulatory elements) and lentivirus-based methods may permit transgenesis in other GINs where it is not currently feasible.

### Mutagenesis and Knockdown in Gastrointestinal Nematodes

Methods for editing endogenous gene expression in GINs remain limited; however, pipelines for targeted mutagenesis using CRISPR/Cas9 were recently established in *Strongyloides stercoralis* and *S. ratti* (132, 135, 136). While difficulties in efficient delivery of genetic material have limited the use of CRISPR/Cas9 in GINs without a free-living phase, two recent studies directly targeted iL3s for mutagenesis. Microinjection of *S. stercoralis* iL3s with CRISPR/Cas9 ribonuclear protein complexes mixed with the liposome-based transfection enhancer lipofectamine yielded mutant phenotypes (137). More recently, CRISPR/Cas9 mutagenesis was achieved in *N. brasiliensis* iL3s through the use of VSV-G-pseudotyped NanoMEDIC EVs loaded with either CRISPR/Cas9 ribonuclear protein complexes or homology-directed repair templates, suggesting that VSV-G-pseudotyped EVs may be a viable strategy for delivery of functional cargo (138). Lastly, although used with great success in *Schistosoma mansonia* (139), RNA interference (RNAi) has been highly inconsistent across GIN species and gene targets (140). However, the precise RNAi induction method used may improve knockdown efficacy (128, 131). Use of short interfering RNA (128, 141) and the recent development of a VSV-G-pseudotyped lentivirus in *N. brasiliensis* offer the hope of achieving more efficient RNAi (134).

Taken together, the recent advances in transgenesis, CRISPR/Cas9 mutagenesis, and RNAi techniques for GINs now enable mechanistic studies of gene function that were previously unfeasible. Future efforts to understand the genetic basis of host–parasite interactions will benefit from additional technical advances, including more efficient generation of stable transgenes or siRNA expression in parasitic adults. Additionally, precise control of transgene expression, both temporal (e.g., iL3 versus adult) and spatial (e.g., internal versus secreted), will be required to interrogate antihelminthic host immunity and immunomodulation by GINs.

## HELMINTH INFECTION AND THE MICROBIOTA

GINs have coevolved not only with their host’s immune system but also with the host’s commensal microbiota, competing for resources in the nutrient-rich intestinal niche (142, 143). This section explains how known intestinal GIN–microbiota interactions govern mutual fitness and host immunological responses.

### Microbiota Effects on Helminth Infection

Some parasitic helminths depend on the microbiota to establish infection. For *T. muris* eggs to hatch, there must be direct contact between intestinal bacteria (e.g., *Escherichia coli*) and the ova, with type 1 fimbriae surface adhesion molecules contributing to this interaction (144). Additionally, *T. muris* can modulate the host intestinal microbiota, selecting for *Bacteroides thetaiotaomicron*, in order to maintain chronic infection (145). While initial infection with *H. polygyrus* is not affected in germ-free mice, survival and fecundity are decreased (146). This fitness defect is due to an expansion of Th2 cells and a corresponding loss of local FOXP3^+^RORγt^+^ Tregs in the absence of the gut microbiome, suggesting that GINs may benefit from microbial-driven immunoregulatory mechanisms. Conversely, the microbiota can help establish resistance to GIN infection via immune-independent mechanisms. For example, microbial production and/or regulation of excitatory neurotransmitters (e.g., acetylcholine) increases intestinal motility to enhance resistance to *H. polygyrus* infection by disrupting larval invasion (147). Taken together, these studies confirm a functional role for the gut microbiota in shaping GIN colonization and fitness.

### Effects of Helminth Infection on the Microbiome and Host Physiology

The immune response induced by GIN infection can also significantly shift the relative abundance and distribution of intestinal bacterial commensals (148–151). In addition to altering the types and abundance of mucins available for bacterial consumption, IL-13/STAT6 signaling in the intestinal epithelium modulates antimicrobial peptide abundance, downregulating *Reg3g* and upregulating *Sprr2a* and *Ang4* (61, 152). Functionally, *T. muris*– or *H. polygyrus*–induced changes in the microbiota limit the expansion of proinflammatory *Bacteroides vulgatus* in a model of Crohn’s disease (153), while *N. brasiliensis* infection reduces segmented filamentous bacteria load and decreases expression of IL-17-associated genes (152). During chronic infection, *H. polygyrus* not only alters the microbiota but also supports a bacterial-dependent increase in the production of immunomodulatory short-chain fatty acids (154) that promote intestinal Treg differentiation (155). Infection with *H. polygyrus* also induces a systemic microbiota-dependent type I interferon response in the lung (156). Additional studies are required to understand the tissue-specific mechanisms by which helminth-induced changes to the microbiota may alter immune responses during both inflammatory disease and infection.

## IMPACT OF GASTROINTESTINAL NEMATODE INFECTION ON HOST IMMUNITY

Numerous observational and clinical studies have found that GIN infection can influence the immune response to various diseases, potentially as a result of the systemic impact of GIN infection. In this section, we call attention to human studies identifying an effect of GIN infection on host immunity to a range of infectious and chronic noncommunicable diseases and link them with animal models that have begun to replicate these effects. In general, however, the development of therapies that selectively replicate the beneficial effects of GIN infection will require a deeper mechanistic understanding of these interactions (for select examples, see the Future Issues section).

### Cross-Protection Against Heterologous Helminth Infection

Endemic distributions of GINs relevant to human health are highly overlapping, presenting the possibility of sequential or concurrent infection with unrelated GINs (3). Importantly, the type 2 effector mechanisms driving helminth expulsion differ between species. IL-4/IL-13-driven goblet cell hyperplasia and intestinal epithelial cell turnover are required to effectively clear *N. brasiliensis* (157–159), whereas mast cells, IgE, and Th2 cells promote *S. venezuelensis* clearance (72). Yet, antihelminth immunity converges on a general type 2 immune response able to provide cross-protection against heterologous GIN infection (Figure 3).

Cross-protection against the hookworm *N. brasiliensis* is not dependent upon the infection route per se; while the threadworm *S. venezuelensis* and roundworm *A. suum* migrate through the skin and/or lung (3, 83, 160–162), the whipworm *T. spiralis* and roundworm *H. polygyrus* are restricted to the intestine (98, 99, 160) (Figure 1). Yet, heterologous protective type 2 immunity appears to act primarily in the skin and lung, not the intestine (3, 83, 98, 99), and the lung plays a dominant role even upon homologous reinfection (95). Cross-protective type 2 immunity between *N. brasiliensis* and *Strongyloides* spp. reduces intestinal adult worm burden, egg production, and fecundity and affects migrating larval morphology and viability (3, 161–163). In this instance, the effect occurs regardless of which helminth is encountered first; lasts for at least 3 months; and requires IL-33, eosinophils, and ILC2s, whereas Th2 cells are dispensable (3). However, prior infection with *S. venezuelensis* does not reduce *N. brasiliensis* intestinal burden when the lung phase is bypassed via oral gavage of adult worms (3). Therefore, protective type 2 immunity reduces intestinal burden and worm vitality before reaching the gut, potentially by retaining and killing larvae in the lung (3, 98, 99) and/or skin, as has been suggested for immune responses upon repeat infection with *N. brasiliensis* (164) or heterologous infection with *H. polygyrus* and *N. brasiliensis* (99, 164). Similarly, *T. spiralis* (98, 160), *A. suum* (83), and *H. polygyrus* (99) cross-protect against secondary *N. brasiliensis* infection, although the relative contribution of innate versus adaptive mechanisms differs between infections.

In keeping with the theme of systemic immunity, intestine-restricted GIN infection can protect against lung stages of a heterologous infection. For example, previous infection with *T. spiralis* leads to ILC2-derived IL-13-mediated upregulation of MUC5B/AC in the lung, thereby trapping *N. brasiliensis* independently of adaptive immunity (98). Conversely, *A. suum* and *H. polygyrus* induce lung-resident Th2 cells that are required for heterologous protection against *N. brasiliensis* (83, 99). These Th2 cells display innate-like functionality and respond to secondary infection in an antigen-independent, ST2/IL-33-dependent manner, indicating that protection is not due to conserved antigens. Lastly, concomitant enteric infection is not necessarily required, as enhanced protection is observed after the primary infection is cleared (98, 99).

Given that cross-protection occurs before intestinal infection, the extent to which heterologous GIN infection protects against strictly enteric helminths (e.g., *H. polygyrus*, *T. spiralis*) remains to be determined. Hyperactivation of the “tuft–ILC2” circuit by *Tritrichomonas* protists confers concomitant resistance to *N. brasiliensis* and *H. polygyrus* infection, suggesting that heterologous GIN infection may function similarly (165). Furthermore, as Th2 cell responses are generated against intestinal worms and acquire innate-like properties in the lung and mesenteric WAT (83, 86), whether intestinal Th2 cells integrate into the tuft–ILC2 circuit requires further investigation. *H. polygyrus*–induced skin Th2 cells are not activated by *N. brasiliensis* antigen, suggesting that cross-protection may rely primarily on innate-like mechanisms in this context as well (85). Lastly, the evolutionary basis for cross-protection remains enigmatic. Some researchers speculate that heightened type 2 immunity to heterologous helminth infection restricts competition for the actively parasitizing helminth (99, 164, 165).

### Protection Against Allergy

Numerous studies and meta-analyses support a general role for GIN infection in suppressing the development of allergic diseases in humans (166, 167). Active infection with *Trichuris trichiura* (168, 169), *Ascaris lumbricoides* (169, 170), *Ancylostoma duodenale*, or other hookworms (167, 170) is associated with a significant reduction in asthma or allergic skin reactivity. Yet allergic protection is not universal and likely depends upon several factors, including the allergic disease, helminth species, infection intensity and status, and age at first infection. Whereas GINs have documented suppressive effects for asthma and allergic skin sensitization (166–170), protection from atopic dermatitis is less clear (171, 172). *A. lumbricoides* infection (166, 167), in contrast to hookworm infection (166, 170, 173), correlates with a higher asthma risk despite reducing allergic skin reactivity, while clearance of *A. lumbricoides* reduces the frequency of asthmatic incidents (174). Hookworm infection strongly correlates with reduced asthma risk when adjusted for infection intensity (166), while anthelminthic therapy diminishes the suppressive effect of GIN infection on allergic skin sensitization (175, 176). Lastly, childhood infection with *T. trichiura* or hookworms is associated with a significant reduction in asthma (169) or allergic skin reactivity later in life (168), respectively; however, experimental infection of adults with the porcine whipworm *Trichuris suis* was less encouraging, producing no measurable improvement in allergic rhinitis symptoms (177, 178). Perhaps the small intestinal tropism of *T. trichiura* and hookworms is particularly important, as *T. suis* predominantly infects the colon.

Regulatory responses induced during GIN infection not only limit antihelminth immunity and associated immunopathology (179) but also contribute to GIN-mediated allergic protection in mice. Tregs play an important role in helminth-mediated allergic protection (100, 180–182). IL-10, produced by Tregs and other immune cells, is necessary for *H. polygyrus*–mediated attenuation of cholera toxin–induced anaphylaxis against peanut extract (180) and OVA:allergen-induced atopic asthma (181, 182). Conversely, diminution of house dust mite–induced allergic airway inflammation by *H. polygyrus* requires Tregs, but IL-10 is dispensable, implicating a role for additional Treg effector functions (100). Expansion of Tregs through *H. polygyrus*–mediated modulation of the gut microbiome also suppresses allergic inflammation (154).

GIN infection additionally influences atopy through Treg-independent mechanisms. Tregs are not required for the *H. polygyrus*–mediated reduction in skin inflammation resulting from dibutyl phthalate–induced contact hypersensitivity; instead, protection likely stems from skin-draining lymph node hypoplasia (103). Interestingly, *H. polygyrus* infection is not protective in an OVA:allergen-induced model of atopic dermatitis (182). Worm products also directly suppress allergic inflammation. HES attenuates allergic airway inflammation by diminishing lung type 2 immune responses, despite preserving Th2 cell differentiation without expanding Tregs (183, 184). Specifically, recombinant HpARI and HpBARI limit IL-33-driven allergic responses (112), while Hp-TGM ablates eosinophilia in both T cell–dependent and –independent models of allergic airway inflammation (185).

### Protection Against Autoimmune Disease

Helminth infection is associated with protection against several forms of autoimmunity, including type 1 diabetes (T1D), inflammatory bowel disease, and multiple sclerosis. Globally, helminth prevalence and T1D incidence are inversely correlated (186). In nonobese diabetic mice, infection before disease onset (~10 weeks of age) with either *H. polygyrus* or *T. spiralis* dramatically reduces the incidence of T1D and destruction of pancreatic β islets (78, 187). Only partial protection is observed when mice are infected at later time points. Strikingly, disease progression is still limited in mice given IL-10R-blocking antibody (78) or genetically deficient in IL-4 (79). Only when the effects of both IL-10 and IL-4 are blocked does prophylactic helminth infection fail to restrict disease progression in nonobese diabetic mice (79). These results suggest that early induction of both regulatory and inflammatory/reparative arms of the antihelminth immune response contributes to limiting T1D progression.

Inflammatory bowel disease arises from environmental, genetic, and microbial factors that instigate a dysregulated intestinal immune response. Initial clinical trials of *T. suis* administration to patients with active Crohn’s disease or ulcerative colitis demonstrated promising, albeit temporary and infection-dependent improvements in symptoms (188–190); however, subsequent trials have observed no significant effect, in part due to remarkably strong placebo effects (191). In mice, several reports have documented both protective and detrimental effects of GIN infection on colitis severity. The DNBS- or TNBS-induced colitis, which emulates environmental inflammatory bowel disease induction, is alleviated by prior *T. spiralis* or *H. polygyrus* infection in an IL-10-dependent manner (192, 193). Furthermore, EVs from *N. brasiliensis* are sufficient to ameliorate TNBS-induced colitis (119). Independently, active inflammation is suppressed and even reversed by *H. polygyrus* infection in two genetic models of colitis: piroxicam treatment of *Il10*^−/−^ mice (194) and transfer of *Il10*^−/−^ T cells into *Rag2*^−/−^ mice (195). In these IL-10-deficient colitis models, suppression of IL-12 and IFN-γ production (194, 195) and induction of tolerogenic DCs by GIN infection were proposed to limit disease severity (195, 196). In the *Nod2*^−/−^ piroxicam model of Crohn’s disease, IL-13/STAT6 signaling induced by *T. muris* or *H. polygyrus* infection protects against the development of intestinal abnormalities (i.e., villus blunting, increased antimicrobial and inflammatory cytokine expression, bacterial translocation) (153). Lastly, *T. trichiura* infection of juvenile macaques improves symptoms of idiopathic chronic diarrhea and is associated with a shift from type 1 to type 2 immune responses in the colonic mucosa and a reduced prevalence of attaching–effacing bacteria (197). These results stand in contrast to microbial-induced colitis, in which *Citrobacter rodentium* exacerbates disease severity in a STAT6-dependent manner upon *H. polygyrus* infection of BALB/c mice (150, 198). Thus, the etiology of inflammatory bowel disease may determine whether type 2 and/or regulatory antihelminth immunity confers protection.

Patients with relapsing-remitting multiple sclerosis who incidentally acquired a helminth infection presented with fewer relapses and reduced disability scores in association with augmented circulating IL-10, TGF-β, and Tregs and a reduction in IL-12- and IFN-γ-secreting cells (199), all of which was reversed following anthelminthic treatment (200). Similarly, a clinical study using *T. suis* therapy for multiple sclerosis observed reduced disease severity and increases in IL-4 and IL-10 during ongoing therapeutic treatment (201). In rodents, establishment of experimental autoimmune encephalomyelitis (EAE) models multiple sclerosis. Infection of rats with *T. spiralis* (202) or mice with *H. polygyrus* (203) ameliorates EAE pathology. Reduced disease severity is associated with increased type 2 cytokine and IL-10 production, reduced IFN-γ and IL-17 production, and Treg induction (202, 203). Notably, Hp-TGM, HES, or chronic infection does not protect against EAE. Instead, protection requires acute *H. polygyrus* infection and *Il4ra* (203), suggesting that early induction of type 2 immunity is the dominant protective mechanism in this model, in contrast to protection against multiple sclerosis, which appears to require chronic infection in humans.

### Protection Against Metabolic Dysfunction

As with T1D, the prevalence of nonautoimmune metabolic dysfunction (e.g., type 2 diabetes mellitus, obesity) is lower in helminth-endemic regions (2). In humans, previous infection with *S. stercoralis* is negatively correlated with incidence of type 2 diabetes mellitus (204). Moreover, concurrent infection with *S. stercoralis* or multiple GINs is associated with elevated levels of serum type 2 cytokines, reduced circulating insulin levels, and increased insulin sensitivity (205, 206), and these effects can be replicated in mouse models. For example, augmentation of the WAT type 2 immune niche following infection with *N. brasiliensis* or *H. polygyrus* confers resistance to genetic and high-fat diet–induced models of obesity (104, 207–209). WAT AAMs, which are sustained by eosinophil-derived IL-4 (104), are crucial for maintaining glucose and insulin tolerance and limiting weight gain when mice are placed on a high-fat diet (210). At homeostasis, WAT macrophages have an alternatively activated phenotype that becomes dysregulated and proinflammatory during obesity (208, 211). Remarkably, enteric helminth infection, which induces WAT eosinophilia (32, 86, 104), reverses obesity-driven proinflammatory reprogramming of WAT macrophages, promoting the appearance of metabolically beneficial AAMs in an IL-4/IL-13- and STAT6-dependent manner (207, 208). Notably, improved metabolic benefits depend on helminth-induced eosinophilia and are sustained long term (104). In contrast, worm clearance in humans reduces the expression of adiponectin, an adipose tissue hormone that regulates insulin sensitivity, and raises circulating insulin levels (206, 212), suggesting that patent infection may be required to sustain beneficial metabolic parameters.

### Altered Susceptibility to Infection

Distinct immunological responses are directed at combating helminth infection (i.e., type 2 and regulatory immunity) versus viral and bacterial pathogens (i.e., type 1 and 17 immunity) and these responses broadly inhibit one another (73). Not only is there extensive overlap in the distribution of GINs and major global pathogens (e.g., *Mycobacterium tuberculosis*, *Plasmodium* parasites), with epidemiological observations suggesting that coinfection alters disease progression (213), but also animal studies have identified modulated responses to viral and bacterial pathogens, principally at mucosal barriers.

Coinfection with *T. spiralis* or *H. polygyrus* increases murine norovirus (MNV) burden in the intestine. This occurs in part because STAT6 signaling impairs the generation of MNV-specific CD8^+^ and CD4^+^ T cell responses (214) but also, in the case of MNV strain CR6, because of MNV tropism for small intestine and colon epithelial tuft cells. Inducing tuft cell hyperplasia with helminth infection or only recombinant IL-4 or IL-25 thus supports MNV CR6 replication (215, 216). More strikingly, mortality induced by the neurotropic flaviviruses West Nile virus, Zika virus, and Powassan virus is exacerbated by *H. polygyrus* coinfection (217). Intestinal motility, epithelial cell turnover, intestinal barrier function, and the antiviral CD8^+^ T cell response are impaired during coinfection, resulting in uncontrolled bacterial dissemination—all of which could be rescued by genetic deficiencies in tuft cells and type 2 immunity.

GIN infection–induced type 2 immunity also impairs antiviral responses beyond the intestine. Coinfection with *A. suum* increases *Vaccinia* virus burden in the lung and enhances weight loss and mortality in comparison to virus alone (218). Exacerbated pathology is associated with a reduction in systemic IFN-γ^+^ CD4^+^ T cells but increased cellular infiltration and inflammatory cytokine production in the lungs, presumably resulting in lethal pulmonary pathology. Human papillomavirus prevalence increases with age in women infected with GINs (107). In mice, prior *N. brasiliensis* infection exacerbates genital herpes simplex virus 2–mediated FRT pathology (e.g., vaginal epithelial ulceration, reduced tissue integrity) (108), consistent with impaired control of herpes simplex virus 2 following IL-33 release in the FRT (219). Simultaneous infection promotes ILC2 and eosinophil recruitment, markedly increasing FRT type 2 inflammation, and depletion of eosinophils is sufficient to abrogate pathology (108). Lastly, systemic reactivation of latent γ-herpesvirus occurs following *H. polygyrus* infection (220). The latent-to-lytic switch is driven by STAT6 binding to the γ-herpesvirus gene 50 promoter, thereby antagonizing the suppressive function of IFN-γ. Notably, a similar cytokine responsive element is present in human γ-herpesviruses.

Concurrent GIN infection also modulates host immunity to pathogenic bacteria and protozoans. Infection of mice with *N. brasiliensis* or *H. polygyrus* impairs clearance and elevates the small intestine burden of *Salmonella enterica* serovar Typhimurium (221, 222). Strikingly, impairment occurs independently of IL-4 and STAT6; instead, *H. polygyrus* induces alterations to the small intestine luminal metabolome, thereby enhancing *S*. Typhimurium pathogenicity (222). In line with observational studies (213), prior infection with *N. brasiliensis* also increases *M. tuberculosis* lung burden (223). Impaired clearance depends upon *Il4ra* and local AAM differentiation, which, compared with IFN-γ-polarized macrophages, display impaired autophagic capability and fail to eliminate intracellular *M. tuberculosis* (224). Finally, simultaneous infection with *H. polygyrus* augments *Plasmodium chabaudi* parasitemia and dramatically increases mortality, whereas protective immunity is restored by worm clearance prior to malaria infection (225). Impaired antimalarial resistance is STAT6 independent and instead is associated with decreased IFN-γ and increased IL-10 and TGF-β expression, implicating a dominant role for helminth-induced regulatory responses in driving exacerbated parasitemia (225, 226).

Notably, helminth coinfection can confer protection against respiratory viruses through various, potentially overlapping mechanisms. Although influenza-specific antiviral T cell responses are impaired by concurrent *T. spiralis* infection (102, 214), coinfection with *T. spiralis* during the enteric, but not chronic muscle, stage of infection protects against influenza-induced pathology (e.g., weight loss, airway occlusion) (227). Protection occurs without reducing viral load and is accompanied by diminished cellular infiltration into the lung and bronchoalveolar space. Reduced inflammatory cell infiltration, potentially a result of impaired mediastinal lymph node responses during GIN infection (102), may explain the amelioration of lung pathology. Comparable observations have been made with respiratory syncytial virus (RSV), where coinfection with *H. polygyrus* reduces RSV-induced pulmonary pathology and proinflammatory cytokine levels while also reducing early viral load (156). Surprisingly, protection is independent of both adaptive and type 2 immunity but instead depends on the commensal microbiome and type 1 interferon induction.

Prior helminth infection can also confer protection against respiratory viral infection. Type 2 remodeling of the pulmonary CD4^+^ T cell and macrophage compartments by previous infection with *N. brasiliensis* enhances SARS-CoV-2 clearance by amplifying the antiviral CD8^+^ T cell response (228). Notably, prior *H. polygyrus* infection is not protective, suggesting a role for tissue tropism. Lastly, both *N. brasiliensis* and *H. polygyrus* infection increase systemic IL-4 (84), which expands virtual memory CD8^+^ T cells capable of contributing to antiviral responses (229, 230). Helminth-induced expansion of murid γ-herpesvirus 4 antigen-specific virtual memory CD8^+^ T cells requires IL-4, occurs independently of cognate antigen, and is sufficient to confer protection against intranasal challenge (229). Thus, a complex interplay of immunosuppression, type 1 interferons, type 2 tissue remodeling, and cytokines can mitigate detrimental immunopathology in the lung in response to pulmonary viral infection.

### Poor Response to Vaccination

Given the ability of enteric helminth infection to modulate the immunological response to infectious agents at peripheral sites, it is unsurprising that numerous reports have documented an impact of GIN infection on vaccination response. Efficacy of vaccination against *M. tuberculosis* (85, 101), *S*. Typhimurium (221, 231), and *Streptococcus pneumoniae* (102) in mice is attenuated by concomitant *H. polygyrus* or *N. brasiliensis* infection. DC migration, antigen-specific T cell expansion, and IgG antibody generation are impaired, but deficits could be restored by helminth clearance or vaccine boosting (85, 101, 221). A meta-analysis of publications describing the effect of helminth infection on vaccination response in humans indicated that infection at the time of vaccination is associated with worse vaccination outcome, a weaker immune response to vaccination, and poorer survival upon pathogen infection (232). Chronic helminth infection in general diminishes vaccination response more than acute infection. Altogether, these observations indicate the need to consider the interplay of helminths and vaccines at several levels, such as (*a*) vaccine design [type (live attenuated, protein, etc.), formulation and immunogenicity (adjuvant), route of administration, cellular versus humoral dependence], (*b*) helminth presence (chronic versus acute, worm clearance, prior or postvaccination infection), and (*c*) vaccination efficacy (humoral and cellular responses, survival with challenge).

## SUMMARY POINTS

1. Nonhematopoietic sentinel cells initiate antihelminth immunity, which converges on a common type 2 immune response that mobilizes effector cells and remodels nonhematopoietic sentinels to function as terminal effectors of parasitic worm clearance.
2. Intestinal helminths directly and indirectly modulate host immunity through (*a*) induction of host resistance and tolerance responses, (*b*) secretion of immunomodulatory products, and (*c*) interaction with the intestinal microbiome.
3. Gastrointestinal nematode (GIN) infection systemically affects the immune tone of peripheral tissues, which can influence both tissue function and disease susceptibility. Helminth eradication has abrogated this effect.
4. Novel tools for genetic manipulation of GINs could permit the dissection of host–parasite interactions that modulate host immunity to other helminths, microbial pathogens, and chronic noncommunicable diseases.

## FUTURE ISSUES

1. How are the effects of intestinal infection transmitted to distal tissues?
2. Can a single worm product, or combination of worm products, substitute for helminth infection with regard to systemic immunomodulation?
3. Do worm-secreted products access the host during lumen-dwelling phases?
4. What is the role of tuft cells in chronic infection and adaptive immunity?
5. What is the role of tissue-resident Th2 cells in heterologous infections or chronic noncommunicable diseases of distal tissues?
6. How do various parameters of GIN infection (e.g., helminth species, infection intensity and status, age at first infection, host resistance/tolerance response, tissue tropism) alter the impact on peripheral immunity and disease susceptibility?
7. When, where, and how do GIN-induced resistance and tolerance mechanisms (e.g., Tregs, IL-10) influence susceptibility to chronic noncommunicable disease?
